# Can Wearable Sweat Lactate Sensors Contribute to Sports Physiology?

**DOI:** 10.1021/acssensors.1c01403

**Published:** 2021-09-22

**Authors:** Kevin Van Hoovels, Xing Xuan, Maria Cuartero, Maarten Gijssel, Mikael Swarén, Gaston A. Crespo

**Affiliations:** † Department of Chemistry, KTH Royal Institute of Technology, Teknikringen 30, SE-100 44 Stockholm, Sweden; ‡ Kinetic Analysis, Sint Janssingel 92, 5211 DA ’s-Hertogenbosch, The Netherlands; § Jheronimus Academy of Data Science, Sint Janssingel 92, 5211 DA ’s-Hertogenbosch, The Netherlands; ∥ Swedish Unit of Metrology in Sports, Institution of Health and Welfare, Dalarna University, SE-791 88 Falun, Sweden

**Keywords:** lactate sensor, sweat, sport, physiology, wearable platforms

## Abstract

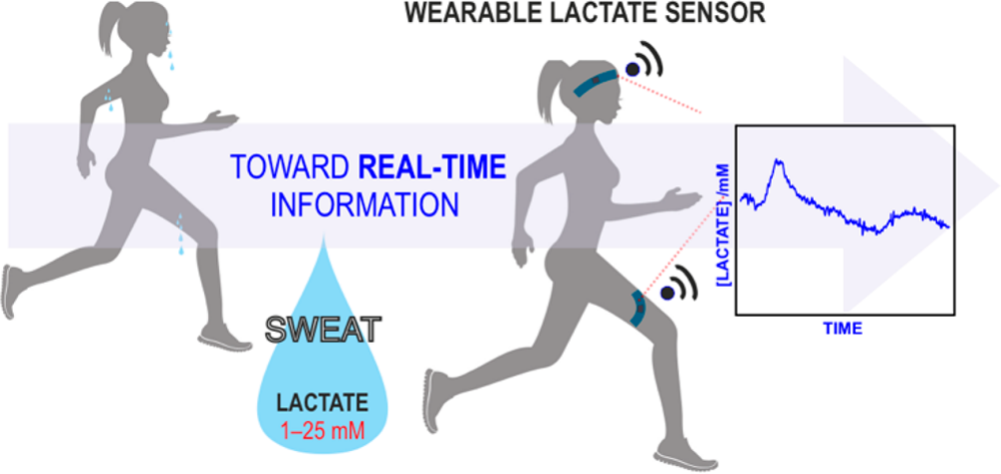

The rise of wearable sensors to measure lactate content in human sweat during sports activities has attracted the attention of physiologists given the potential of these “analytical tools” to provide real-time information. Beyond the assessment of the sensing technology *per se*, which, in fact, has not rigorously been validated yet in controlled conditions, there are many open questions about the true usefulness of such wearable sensors in real scenarios. On the one hand, the evidence for the origin of sweat lactate (e.g., via the sweat gland, derivation from blood, or other alternative mechanisms), its high concentration (1–25 mM or even higher) compared to levels in the blood, and the possible correlation between different biofluids (particularly blood) is rather contradictory and generates vivid debate in the field. On the other hand, it is important to point out that accurate detection of sweat lactate is highly dependent on the procedure used to collect and/or reach the fluid, and this can likely explain the large discrepancies reported in the literature. In brief, this paper provides our vision of the current state of the field and a thoughtful evaluation of the possible reasons for present controversies, together with an analysis of the impact of wearable sweat lactate sensors in the physiological context. Finally, although there is not yet overwhelming scientific evidence to provide an unequivocal answer to whether wearable sweat lactate sensors can contribute to sports physiology, we still understand the importance to bring this challenging question up-front to create awareness and guidance in the development, validation, and implementation of wearable sensors.

We have witnessed the growth of wearable chemical sensors as a promising decentralized concept capable of digitizing levels of relevant chemical targets in diverse human fluids. The ultimate aim of these devices is to provide real-time information on the health or athletic status of the patient/user. It is worth noting that a large proportion of the research efforts dedicated to the development of wearable chemical sensors has mainly been focused on sweat monitoring, with extraordinary contributions from eminent groups worldwide.^1−8^ This is, in fact, perhaps not surprising as sweat collection is accessed through noninvasive procedures, and it also presents a large variety of analytes from which to obtain physiological information. Sweat does not only contain water (99%) but other constituents such as electrolytes (e.g., sodium, chloride, potassium, and bicarbonate ions), metabolites (such as glucose, urea, ammonia, ethanol, and amino acids), and micronutrients (iron, magnesium, calcium, copper, zinc, and vitamins).^9^

To date, one of the main concerns in sports physiology research is to understand whether sweat content could be used to monitor the performance and hydration status of the subject under study.^10^ It is evident from the literature that research has mainly focused on targeting sodium (Na^+^) and chloride (Cl^–^) ions, which reflect hydration status,^10^ but also, and perhaps more recently, there is a remarkable interest in sweat lactate.^11−14^ There is believed to be a connection between sweat lactate and exercise intensity and that this therefore could be used as a proxy to tedious blood lactate measurements.^11,12^ Similarly, there is a set of clinical biomarkers, mainly present in blood and to a lesser extent in urine, associated with metabolic health, hydration and muscle status, endurance performance, injury status and risk, and inflammation as well (see the review by Lee et al. for more details about the nature and relationship of such biomarkers).^15^ More specifically, blood lactate measurements are used during field and lab testing to trace effort, determine lactate threshold together with training zones, and provide training advice for the athlete.^16,17^ However, blood collection for further lab-based analysis is traditionally accomplished through invasive punctures, which are sometimes painful and even cause discomfort to the subject and/or disturb the sports practice. Moreover, the entire procedure (collection and analysis) is unsuitable for continuous measurements: discrete traces are provided with some delay after the sporting activity has ended.^18^

Sweat lactate sensors have been proposed to be a promising solution to overcome the typical drawbacks of most blood tests. Moreover, sweat has been claimed as the source of next-generation digital biomarkers for medical diagnosis among cystic fibrosis, kidney failure, lung cancer, and Parkinson’s disease, among others.^19^ Related to lactate sensing, there is vivid debate on the potential relationship between sweat and blood lactate^13,14^ and, in general terms, about the different pathways that result in a mixed generation or modulation of lactate levels in the body. In the past, blood lactate was considered a waste product that impaired sports performance.^20^ However, over the last 30 years, it has been demonstrated that lactate has different roles in the body and possesses a crucial role during certain physical activities. The three major known physiological functions of lactate are as follows: (i) lactate is the major energy source in the body (optimal fuel for working muscles), (ii) lactate is a gluconeogenic substrate, and also (iii) lactate is a cell signaling molecule.^21^ On one hand, the so-called “lactate shuttle” concept is related to the role of lactate in the delivery of oxidative and gluconeogenic substrates as well as in cell signaling (Figure 1).^21^ On the other hand, muscle lactate production was reported to be essential to improve exercise performance, which was concluded through blood lactate measurements taken in parallel to an evaluation of the individual’s performance.^21,22^

**Figure 1:**
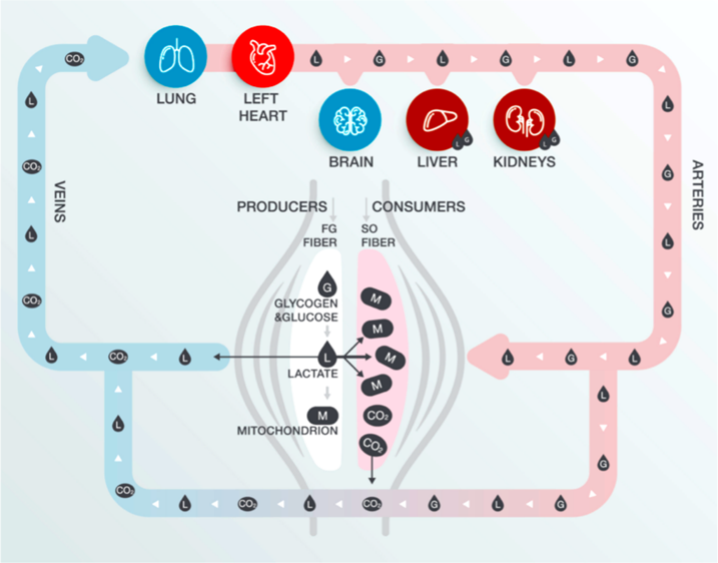
Illustration of the lactate shuttle in blood. Cell–cell and intracellular lactate pathways describe the roles of lactate in the delivery of oxidative and gluconeogenic substrates as well as in cell signaling. G = glucose and glycogen; L = lactate; M = mitochondrion. CO_2_ = carbon dioxide.^21^ Reproduced with permission from ref (21). Copyright 2018 Elsevier.

In contrast to the well-understood “lactate shuttle” in blood, the mechanism and physiological pathway of lactate in sweat still remain unclear.^9^ The first observations of lactate in sweat were made by Schenk and Wissemann in 1926.^23^ Since then, there have been many discussions about the origin of lactate in sweat. Until very recently, researchers believed that lactate in sweat is not derived from the blood because of the significant difference in concentration between lactate levels in these two fluids and due to the unclear relationship between blood and sweat concentration during a physical practice (e.g., regular exercise).^9,10,13,14,24,25^ This difference was then assumed to be related to the lactate production of the eccrine sweat gland itself.^9,25^ Subsequently, recent studies highlighted a relationship between blood and sweat lactate,^12,26−34^ which reinforces the hypothesis of another mechanism for the appearance of lactate in sweat. For example, it has been proposed that the increase in lactate production from muscle cells may induce a simultaneous rise in blood and sweat lactate through a change in autonomic nervous balance, hormones, acid–base equilibrium, and metabolic dynamics.^12,21^

Parallel to the case of lactate levels, sweat glucose is very low (0.01–0.2 mM) compared to blood glucose (4–10 mM),^10^ and some papers have claimed strong correlations between sweat and blood glucose despite the large difference in the concentration of the two fluids.^26,35,36^ In contrast, other authors did not observe such correlations.^14^ Thus, there is a tangible controversy in the field, likely arising from the fact that there are no universally accepted approaches for sweat collection and analysis that provide reliable data to enable the identification of a clear correlation with blood. Additionally, the results strongly depend on environmental conditions and the biological pathway for the subject to perspirate. Considering this dilemma and lack of schematization of glucose measurements, which is the most analyzed biomolecule in recent centuries, it might be expected that a similar situation (and even greater ignorance) exists for lactate and other compounds. One can find published papers in the literature with incisive titles, such as ‘*The (in)dependency of blood and sweat sodium, chloride, potassium, ammonia, lactate and glucose concentrations during submaximal exercise*’,^10,14^ which clearly signals the need for more clarity in the field.

The main aim of this perspective paper is to provide our understanding of the current panorama of sweat lactate sensing and discuss its usefulness in sports physiology. We have considered opinions from scientists actively working in the domains of wearable sensors as well as sports physiologists. Accordingly, we consider it necessary to start with an evaluation of the available methods for human sweat collection, as this is related to the reliability of the lactate observation and, hence, the derived physiological conclusions. Next, we analyze the current state of electrochemical lactate sensors integrated in wearables, and we list key features to be improved or changed toward the final success of the technology. We also comment on other techniques available for tracing the “threshold” in sports performance. Future research is undoubtedly necessary to investigate the mechanism of lactate production, and it is likely that the use of wearable chemical sensors could be a potentially useful analytical tool to shed light onto this issue.

## Methods to Collect Human Sweat

To provide accurate sweat analysis measurements, the research community should strive not only for the right analytical assessment and validation of the detection technique but also for the appropriate selection of the sample collection method. Most of the studies showing relevant physiological information to date are based on a pure sweat collection approach followed by a lab-based analysis. Indeed, researchers are still trying to improve the existing procedures and develop new sampling methods to ensure that the chemical and physical integrity of the sweat sample is maintained during its collection, while also handling the analytical characterization.^37^

To the best of our knowledge, the first investigations related to sweat analysis date back to the 1930s. In these studies, the researchers used the so-called whole-body washdown (WBW) technique for sweat collection.^38−40^ In this technique, the subject is placed inside a plastic isolation chamber where he/she practices a sport (e.g., biking). The subject is washed with deionized water before and after exercise. All the equipment and clothes that the subject touches during the experiment are placed in the bottom of a silage bag (along with the sweat and wash solution). After thoroughly mixing the contents collected at the bottom of the silage bag, a sample (postwash) is collected for later analysis. The WBW method was initially considered the most accurate technique for whole body sweat collection, because all the sweat that the subject loses is collected and it does not interfere with the normal evaporative sweating process. However, the approach requires a very controlled setting, it has a certain level of invasiveness and, because it accounts for sweat loss, sweat content refers to average body concentrations rather than local observations.

**Figure 2:**
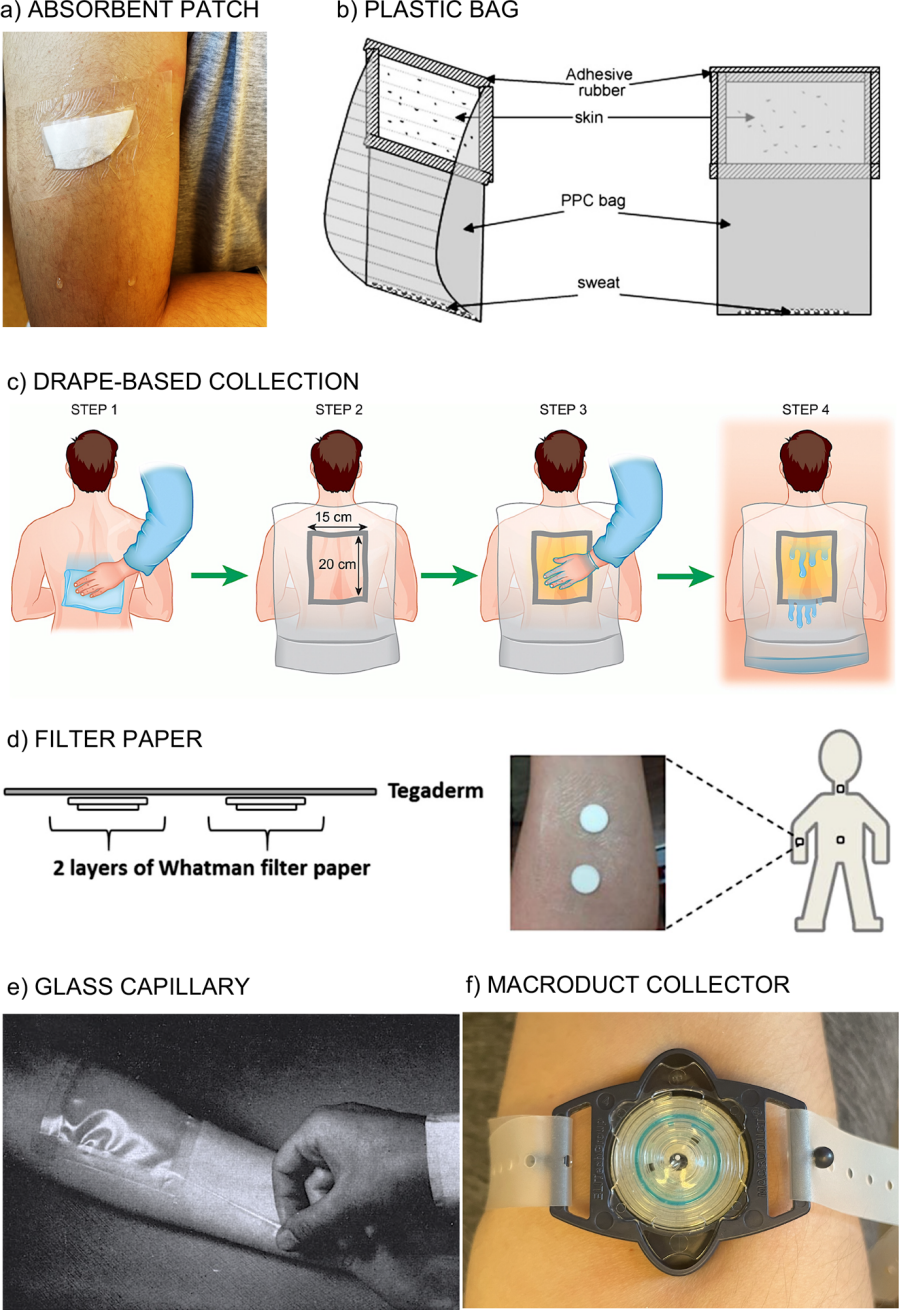
(a) Absorbent patch based on a cotton pad fixed with hydrofilm tape.^45,46^ (b) Native sweat collecting system created from polypropylene copolymer bag and adhesive rubber: a window covers a specific skin area, and a “bag part” allows sweat to accumulate. Reproduced with permission from ref (48). Copyright 2007 Elsevier. (c) Drape-based collection.^49^ Step 1: The whole back area is wiped with tap water. Step 2: A drape with a square hole is fixed. Step 3: Petrolatum is applied to close the hole. Step 4: The bottom part of the drape is folded up to create a pocket for fluid collection while subjects are sweating in a sauna at 80 °C. Reproduced with permission from ref (49) under a Creative Commons Attribution (CC BY) license. (d) Skin patch based on filter paper for sweat collection. Reproduced from ref (51) (https://pubs.acs.org/doi/10.1021/acs.analchem.7b01988). Copyright 2017 American Chemical Society. (e) Glass capillary introduced in a closed plastic environment for collection of stimulated sweat.^52^ Reproduced with permission from ref (52). Copyright 1965 Elsevier. (f) Macroduct operating for sweat collection after iontophoresis is applied.^56^

The local collection of sweat directly from the skin surface has been proposed in the subsequent years.^39,41−43^ The collection might be accomplished by means of absorbent patches (Figure 2a),^39,44−46^ plastic bag (Figure 2b),^47,48^ clothes and fabrics (Figure 2c),^49^ filter paper (Figure 2d),^50,51^ glass capillaries (Figure 2e),^52^ cotton gloves/socks,^53^ Parafilm-M pouches,^50^ latex gloves, or the Macroduct collector (Figure 2f).^54^ Despite the plethora of possibilities, the definition of each technique *per se* may already alert the reader (and possible user) about a series of drawbacks regarding the maintenance of the sweat integrity. In the following, advantages and drawbacks of the most common sweat collection methods are discussed.

Absorbent patches (Figure 2a) possess a hydrophilic and porous structure that is fixed directly on the skin and covered by an outer membrane or material that attaches to the skin to try to avoid evaporation. The entire patch configuration is flexible, lightweight, low cost, and surface adaptable and can be used in one or more specific collection sites (i.e., different parts of the body). Cleaning the skin (e.g., with distilled water, soap, and a surgical scrub brush) is recommended to reduce contamination of the collected sweat.^55^ Indeed, a cleaning process is always indicated independent of the collection method to avoid large contamination issues and ensure the good adherence of the elements involved in the collection device to the skin (see Figure 2c as an example).

In particular for the absorbent patches, the results are known to be affected by volume loss, regional sweat variation, health state, exercise intensity, and environmental conditions.^44^ For example, volume loss varies across the body and may show differences of up to 360% at different collection sites. Also, some diseases cause a higher production of sweat, while others inhibit it. Additionally, higher exercise intensity produces more sweat, and environmental conditions (temperature and humidity) are also known to influence sweat rate. Taken together, these variables may result in the saturation of the absorbent patches and hence affect the results.^43^ Some other drawbacks are related to the relatively long time that is needed to prepare and replace the patches depending on the configuration of its elements, the risk of partial/total skin detachment (high evaporation risk), and also the possibile effect of hydromeiosis on sweat rate.^57^

Plastic bags placed on the arm (Figure 2b) have been used for a long time as a simple and disposable sweat collection option. Indeed, in 2007, Appenzeller et al. developed and commercialized a system consisting of an adhesive rubber to cover the skin and a polypropylene copolymer bag to accumulate sweat.^48^ While the average local sweat rate can be calculated from pre/postexercise pad weights, the collection system *per se* might modify the local skin environment in which it is fixed and, therefore, alter the flow rate of sweat onto the skin surface. Moreover, the occlusive skin covering causes a lack of ventilation and increases moisture accumulation on the skin, which leads to progressive blocking of sweat ducts and sweat suppression.^43^ In constrast, other methods have claimed to avoid such occlusion issues, such as collectors based on filter-paper material (Figure 2d).^51^

One of the oldest methods is based on sweat collection via scraping, dripping, or gathering it from the exposed skin into a capillary, test tube, or beaker (see Figure 2e).^52^ While this method is evidently prone to many sources of potential error, especially due to evaporation and contamination issues, it has certainly helped progress the evolution of sweat characterization. Because of the emergence of more advanced collection protocols, the method is no longer recommended for sweat collection.^37^

Today, the Macroduct collector (Figure 2f) is commonly used in sweat research, as it is probably the most evolved solution for sample collection.^56,58^ It was initially devised for cystic fibrosis diagnosis in newborns as part of a clinical device that combines chemical stimulation of sweat via iontophoresis with pilocarpine, but it can also be used with natural perspiration.^54^ In essence, the collector part consists of a spiral-shape flexible tubing mounted in a plastic base with a hole coinciding with the inlet of the tubing. The plastic base, in turn, contains a strap that permits the attachment of the device to the arm with the hole coinciding with the place in which the iontophoresis was exactly applied (Figure 2f). It is easy to visualize that the collector is working properly because of a blue dye that provides color to the sweat entering the tubing. The sample is finally extracted from the collector with a syringe and later analyzed.

Because sweat is immediately removed from the skin, the Macroduct collector can avoid contamination, leakage, and hydromeiosis.^57^ However, the final step of sweat harvesting with the syringe is rather delicate, and inadequate handling may cause the entire sweat volume (ca. 40 μL) to be lost. On the other hand, the collector could be attached to any part of the body as long as the strap size is redesigned to allow for convenient fixation. We have indeed successfully tried this in the forehead, arm, back, and thigh. In our opinion, the operation of the Macroduct collector may have three main roles in the future development of wearable sensors, including lactate detection. First, owing to the blue dye, it is possible to follow the perspiration rate in the individual via imaging.^54^ Second, the collected sample could be used to validate the accuracy of on-body measurements. Third, the outlet of the tubing could be coupled to a wearable sensing device to provide continuous sweat measurements.

In general terms, the sweat rate and content measured by all of these local methods for collection are highly dependent on the body site and are not at all representative of the sweat content and average sweat rate of the whole body.^10,43,59^ There appear to be large differences between regional sweat rate compared to the whole body average: from 0.5 mg cm^–2^ min^–1^ at the forearm to 2.4 mg cm^–2^ min^–1^ at the forehead and 0.7 mg cm^–2^ min^–1^ on average for the whole body. Sweat lactate concentrations also range from 6.5 mM in the forehead to 13 mM in the foot, with an average concentration of 5.9 mM for the whole body.^59^ In principle, and indeed depending on the true mechanisms involved in sweat lactate production/consumption, it is believed that sweat rate influences the lactate composition in terms of a “dilution effect” with increased rates of sweating. Nevertheless, lactate production could be body-region-dependent because of the different implications of active and passive muscles in physical activity.^33^

According to our own experience operating with WBW and the majority of local sweat collection methods, the conclusion is that the available procedures are complicated and require trained researchers. In addition, the obtained sample volumes are usually very low (a few microliters) and not sufficient for reliable handling to perform analytical measurements using centralized lab equipment. Furthermore, the risk for analyte degradation and sample evaporation is relatively high in all the methods.^2^ One strategy to be adopted is a wearable device, in which the sweat collection is completely isolated from the environment but also allows for natural perspiration and no blocking of the sweat glands when worn by the individual. At the same time, the sweat should flow inside the device in such a way that the sample is measured by the analytical machinery and is continuously replaced at a similar speed to the subject’s sweat rate, providing close to real-time and continuous on-body measurements. Sports physiology will likely benefit from a technology able to account for high-resolution temporal lactate changes according to the intensity of the physical activity, rather than discrete information from centralized lab-based analysis. Otherwise, the information on which the scientific community is grounding physiological interpretations and statements would never be accurate enough to elucidate the role of lactate in the body, particularly considering the sports domain.

## Wearable Sweat Sensors

Wearable devices for sweat characterization in individuals have been suggested as the technological solution to address the open and future questions in sports physiology. This applies to analysis of not only lactate but also other biomolecules (mainly glucose) and ions.^46,60−62^ Systems enabling a sweat-flow analysis have been demonstrated and are based on different strategies such as absorbent materials, surface wettability combination, and advanced microfluidic systems.^37^

### Absorbent Materials

**Figure 3:**
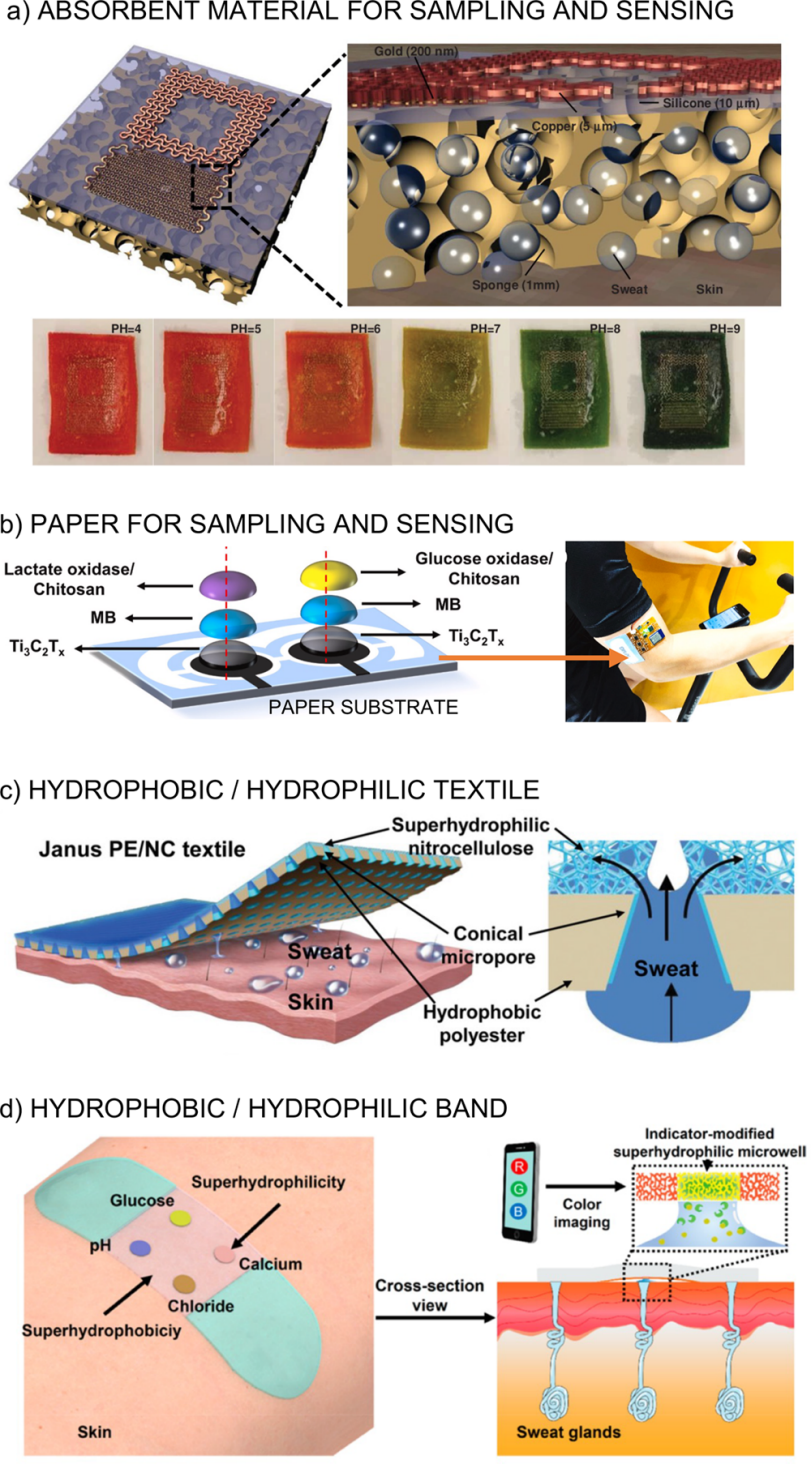
**(a) Top:** A wireless capacitive/colorimetric sensor designed to be attached to the skin. It can be seen how Cu^2+^ accesses the sensing zone. **Bottom:** Change of color at different pH levels in sweat. Adapted from ref (64). Copyright 2014 John Wiley and Sons. **(b) Left:** Modification of the paper substrate to obtain glucose and lactate sweat sensors. **Right:** On-body tests using the paper-based wearable sensors. Adapted from ref (65). Copyright 2021 Elsevier. **(c) Left:** Sweat output pathways of the human body covered with the hydrophobic/hydrophilic textile. **Right:** Conical micropores across the textile designed to transport sweat by asymmetric curvature. Reproduced with permission from ref (72). Copyright 2019 John Wiley and Sons. **(d) Left:** A sensing band worn on the subject’s skin. **Right:** Cross-sectional view of sweat pumped from subcutaneous sweat glands and precisely attracted onto indicator-modified superhydrophilic microwells for colorimetric cetection. Reproduced from ref (73). Copyright 2019 American Chemical Society.

Absorbent materials have been widely used in the sweat sampling zone in wearable sensors.^3^ Paper, nonwoven fabrics, cellulosic materials, hydrogels, and rayon pads have been shown to efficiently collect and guide sweat from the skin to the sensing surface.^63^ Importantly, absorbent materials can also be functionalized with sensing components and thus serve a dual purpose of both sampling and sensing. The Rogers group reported on a wireless patch for colorimetric and/or capacitive ion sensing (OH^–^, H^+^, Cu^+^, and Fe^2+^) by a hydrophilic porous substrate coveniently modified to provide interdigitated electrodes while sampling the sweat through capillary forces (Figure 3a).^64^ Li et al. recently investigated highly integrated sensing paper for real-time amperometric determination of glucose and lactate in sweat.^65^ This device is able to transport and accumulate sweat on the electrodes’ surfaces, which are patterned in the paper substrate and connected to an electronic board for on-body measurements (Figure 3b). Colorimetric detection of glucose by a paper-based electrode connected to a cotton thread that conducts sweat from the skin to the electrode has also been reported.^66^ Advantageously, the thread can be obtained from any sports clothes and material.

While many different approaches in a similar vein can be found in the literature, they all aim to generate an average analysis of the sweat that accumulates between the skin and the sensing surface while perspiring, rather than providing a continuous characterization of newly generated sweat.^67−69^ It is difficult to envision that absorbent materials are able to provide a true sweat-flow system encompassing perspiration.

### Superhydrophobic/Superhydrophilic Surfaces

Interestingly, sweat transport can be achieved via materials that combine hydrophobic and hydrophilic properties.^70−72^ Dai et al. reported on a hydrophobic/hydrophilic textile with asymmetric micropores for efficient sweat transport.^72^ When the sweat makes contact with the hydrophilic micropores, the textile is able to pump sweat to the superhydrophilic layer by capillary force, as depicted in Figure 3c. On the other hand, double-face knitted fabrics in which one side is hydrophobic and the other side is hydrophilic have been successfully used for the fabrication of sports clothes with moisture management properties and thus control of thermal absorptivity, which determines the warm-cool feeling of the cloth.^72^ Beyond this application, the potential of this class of materials to be combined with chemical sensors for sweat analysis is paramount.

Zhang and co-workers reported a flexible band that combines superhydrophobic–superhydrophilic microarrays with nanodendritic colorimetric biosensors for sweat sampling and in situ analysis of pH, glucose, calcium, and chloride (Figure 3d).^73^ In essence, the superhydrophobic substrate is able to confine microdroplets into superhydrophilic microwells. On-body investigations have revealed that the secreted sweat is repelled by the superhydrophobic area and precisely collected and sampled onto the superhydrophilic micropatterns. However, it is necessary to demonstrate the utility of hydrophobic/hydrophilic materials to provide a constant and renewed sweat flow via implementation of clear inlets and outlets in the wearable device. We foresee greater possibilities with the use of hydrophobic/hydrophilic materials than with traditional absorbent materials.

### Sweat Flow Assisted by Polydimethylsiloxane

**Figure 4:**
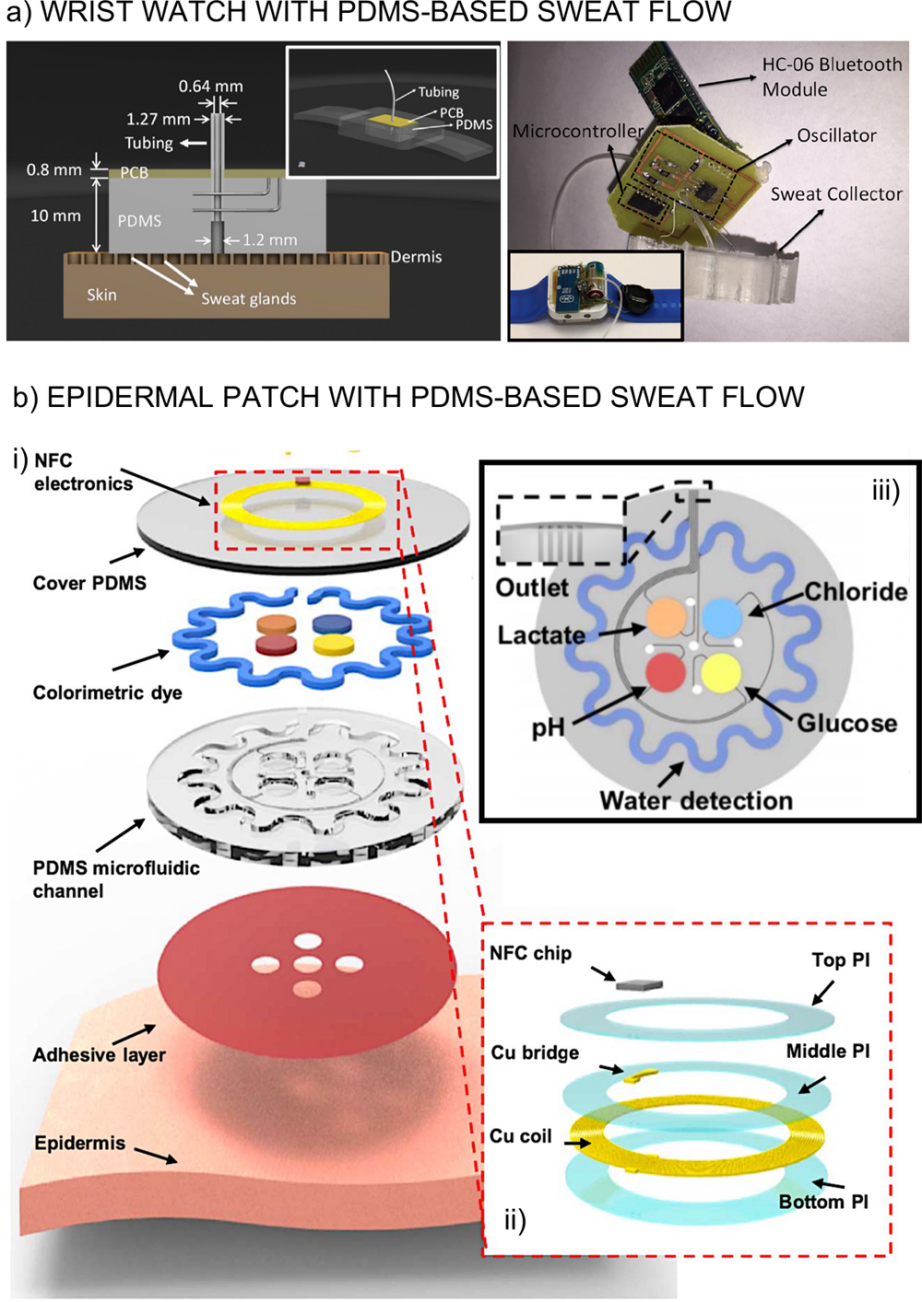
**(a) Left:** The PDMS sweat collector including the conductivity detector. Inset: Design of the wrist watch including the PDMS sweat collector and PCB board. **Right:** Real pictures of the device. Reprinted with permission from ref (74). Copyright 2016 Elsevier. (b) Layering of a wearable device including the PDMS microfluidic channel **(i)**. Magnification of the electronic system **(ii)**. Microfluidics involving five channels: four for colorimetric sensing and one for the sweat rate measurements with the corresponding inlets and outlets **(iii)**. Adapted from ref (75). Copyright 2016 American Association for the Advancement of Science.

Sweat sampling and flow have been demonstrated to be possible by means of microchannel patterning. Initial attempts were mainly based on the use of polydimethysiloxane (PDMS) material.^74^ One interesting example is the wrist watch presented by Kaya and co-workers in 2016, based on a PDMS sweat collector functioning via hydraulic pumping action of sweat glands after appropriate sealing with the skin.^74^ As illustrated in Figure 4a, the PDMS structure directly touches the skin and allows sweat glands to secrete the sweat through the collection hole and the connected tubing. As the sweat goes into the system, it passes through two wires which are connected to the PCB board to measure the conductivity of the sweat, which is supposed to account for changes in the total electrolyte content in the sweat. The main problems arising from the on-body usage of this device seem to come from poor sealing between the PDMS material and the skin, which is indeed something to be considered in all collectors discussed here. In our opinion, the prototype by Kaya and co-workers may benefit from the utilization of clinical grade tape. A further adaptation for chemical sensing would involve the redesign of the device to be adapted for different sensor configurations.

The approach introduced by the Rogers group, also in 2016, deserves special attention.^75^ The device possesses a series of capabilities which may provide highly insightful directions toward developing effective microfluidic devices for sweat analysis. A PDMS structure was designed in such a way to define five small areas to provide sweat access. Then, each of these five areas is connected to a microfluidic channel that goes either through an optical reservoir for the colorimetric readout of pH, chloride, glucose, and lactate or through a serpentine containing a blue dye. Each channel ends in an outlet. A schematic illustration of the wearable prototype is shown in Figure 4b. The sweat flows through the PDMS channels owing to a combination of capillary action and the natural pressure associated with perspiration. Importantly, backpressure problems are minimized due to the design of every channel. The dye in the sepertine channel then allows for a correction of the measurements toward close to real-time profiles. However, the colorimetric detection method is the real impediment toward continuous measurements, and therefore only discrete on-body profiles were demonstrated.^75^ The redesign of the microfluidic system to host electrochemical sensors rather than colorimetric ones could elevate the operation of the prototype to the next level regarding the acquisition of high-resolution data.

### Last Generation of Microfluidic Devices for Sweat Analysis

Recent advances in manufacturing techniques have served as a strong foundation for the development of wearable analytical devices that combine the sensing part, microfluidics, and electronics into “skin-like” on-body sensing strategies.^60,76−78^ The Javey group presented in 2018 a highly integrated device for the electrochemical detection of potassium, sodium, glucose, and lactate combining potentiometry and amperometry in a very miniaturized wrist band.^26^ In a similar direction, Pirovano et al. reported the SwEatch platform in 2019–a wearable sensor for sampling and measuring the concentration of sodium and potassium in human sweat with a high level of integration.^79^ However, again, this sort of wearable device works on the basis of sweat accumulation, and thus, its final application would be more suited to clinical applications because of the impossibility of accounting for temporal concentration changes. Indeed, to the best of our knowledge, it is difficult to find wearable devices that properly function on a microfluidic basis to analyze perspiration, whereas accumulation-based sensors are widely reported in the literature.^11,69,80^

**Figure 5:**
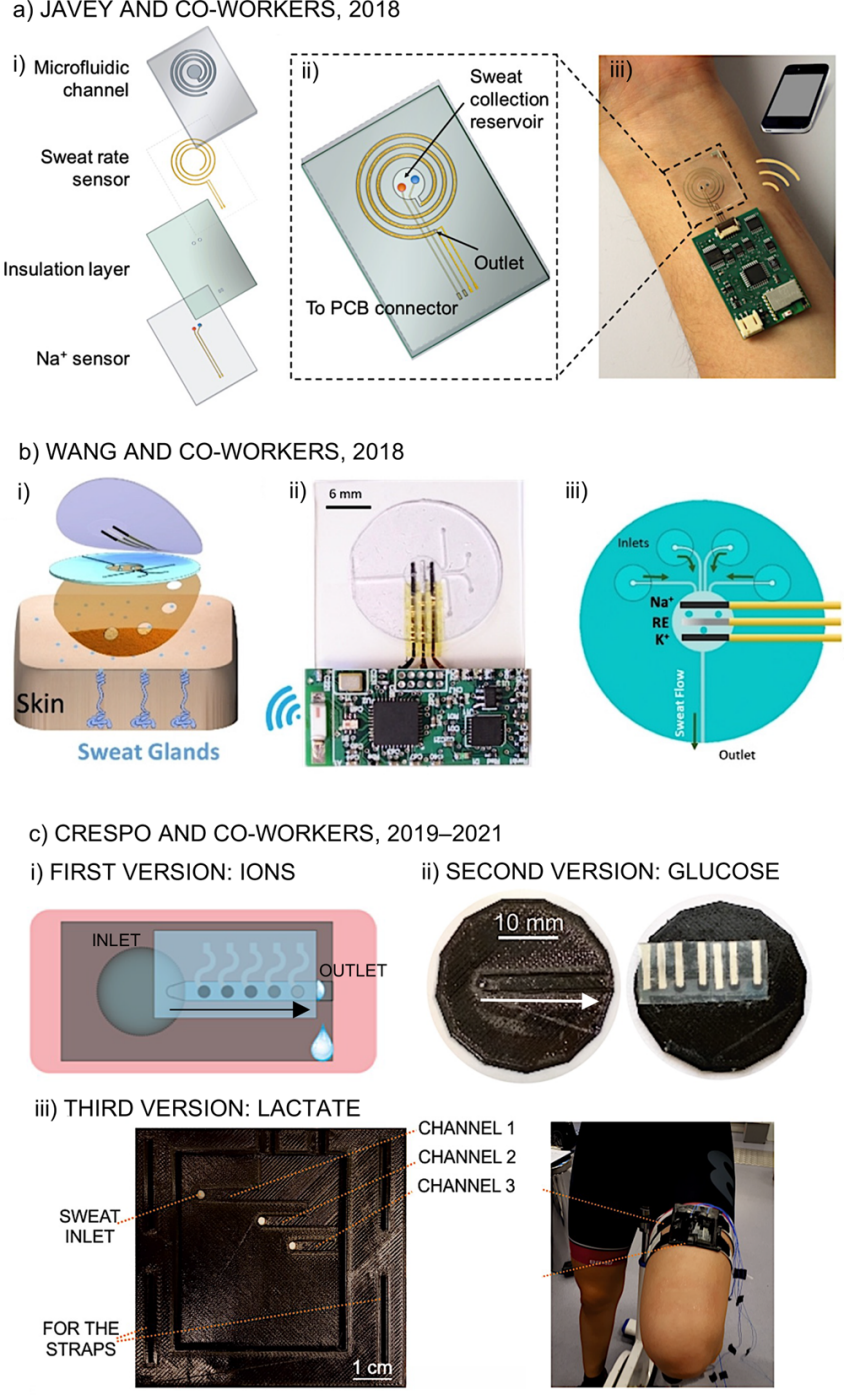
(a) A wearable sweat-sensing patch composed of four layers: a spiral-patterned microfluidic channel, a pair of parallel Au electrodes for sweat rate sensing, a parylene-C insulation layer, and ion-selective electrodes **(i)**. A complete view of the device **(ii)**. Real picture of the device worn on the user’s wrist **(iii)**. Reproduced from ref (60). Copyright 2018 American Chemical Society. (b) Assembly of the parts of a wearable device on top of the skin, in this order: skin adhesive tape, microfluidics (in PDMS), and sensors (patterned also on the PDMS substrate) **(i)**. Real image of the wearable device including electronics **(ii)**. Schematics of the fluidics operation **(iii)**. Adapted from ref (81). Copyright 2018 John Wiley and Sons. (c) Wearable devices with the sensors embedded in the microfluidic channel. First design proposed for detection of ions **(i)**. Reproduced from ref (45) (https://pubs.acs.org/doi/abs/10.1021/acs.analchem.9b02126). Copyright 2019 American Chemical Society. Second design developed for glucose analysis **(ii)**. Reproduced from ref (46) (https://pubs.acs.org/doi/10.1021/acs.analchem.0c02211). Copyright 2020 American Chemical Society. Third (and more advanced) prototype for lactate sensing **(iii)**. Reproduced from ref (78) (https://pubs.acs.org/doi/abs/10.1021/acssensors.1c01009). Copyright 2021 American Chemical Society.

In that context, the Javey group reported in 2018 a wearable sensor based on a microfluidic channel that enhances real-time electrochemical sensing of ions together with the sweat rate.^60^ The microfluidic component was comprised of a spiral-patterned patch in which potentiometric ion-selective electrodes and electrical impedance-based sweat rate sensors are embedded, as shown in Figure 5a. Progressive sweat flow was demonstrated to be governed by the pressure induced by the secreted sweat. However, while the size of the circular sweat reservoir can be modified with a different number of electrodes targeting different ions, the size of this element is crucial to provide truly real-time profiles.

The higher the area (and thus the volume) of the sweat reservoir, the longer the time required to completely fill it, and thus the start of the passive sweat flow through the microfluidic channel. Overall, the sweat will be mixed in that sweat reservoir, and the provided measurements (analyte concentration) will be an average of the accumulated sweat. The observed dynamic concentration profiles will be close to, but never exactly, real-time tracing. On the other hand, and in the same direction as the device previously reported by Rogers and co-workers,^75^ sweat rate measurements (and the volume of the sweat reservoir) may allow implementation of an algorithm for the dynamic correction of the readout.

In the same year, Sempionatto et al. presented a wearable device based on a microfluidic configuration that allows efficient natural sweat pumping to a potentiometric detection chamber containing potentiometric ion-selective electrodes for sodium and potassium.^81^ In contrast to the design proposed by Javey and co-workers,^60^ the microfluidics is composed of a series of small inlets connected to channels that direct the sweat into a circular detection chamber containing the sensors (Figure 5b). When this chamber fills, the sweat is then naturally replaced, pushed by the subject’s perspiration accessing the inlets. Again, the volume of the chamber will determine how close these measurements are to the real-time scenario. Interestingly, the paper also shows a series of on-body measurements.^81^

In a different direction, since 2019, Crespo and co-workers have presented a series of 3D-printed devices in which the electrodes are embedded in the same microchannel into which the sweat flows after collection from the skin.^45,46,78^ The first version of the wearable device was based on a circular inlet whose diameter was slightly greater than the channel height to ensure passive sweat flow while the subject is perspiring (Figure 5c, part (i)).^45^ The channel contained separate potentiometric ion-seletive electrodes for chloride, potassium, sodium, and pH detection, which were positioned one after the other in the channel. Unfortunately, the first true measurement that the device provides only comes once the sweat reaches the reference electrode (15–20 min), which is placed in the last position of the channel. In this work, the attention devoted to the validation and demonstration of the accuracy of the on-body measurements is important.^45^

The second version of the wearable device was focused on sweat glucose sensing and comprises a circular inlet inside the channel, with a diameter lower than the height of the channel (Figure 5c, part (ii)).^46^ After a thoughtful investigation of the influence of several factors in the electrode response, the glucose sensor was accompanied by pH and T sensors to correct the observed readout. Again, a delay in the availability of the first data points during the practice was experienced as a result of all the necessary sensors being placed in sequence in the microfluidic channel. Advantageously, the third version of the prototype (Figure 5c, part (iii)) was based on separate microfluidic channels for each analyte (i.e., lactate, pH, and T), thus improving the response time of the device.^78^ The wearable was also redesigned to improve other capabilities, such as portability on any part of the body (forehead, arm, back, thigh), sweat dissipation, and higher electronics compatibility. Overall, avoiding having chambers/reservoirs for sweat collection and/or detection seems to be the direction to follow for dynamic profiles that are closer to the real-time situation in the body. Moreover, the integration of a strategy able to provide the sweat rate not only inside the wearable but also in the area scrutinized in the subject may allow for a “double” dynamic correction in the observed readout, enabling the collection of even more accurate results.

## Wearable Sweat Lactate Sensors

Having discussed the strategies to provide sweat collection and sweat flow to reach the sensor surface, we now focus on the specific case of lactate detection. Based on the established conclusions and our own experience in the field, the following key features should be considered to achieve a reliable wearable sensor for continuous lactate detection in sweat: 1) the sampling method must encompass the subject’s perspiration and avoid any sweat evaporation and contamination risk once fixed on the skin; 2) the device is fixed to the skin to provide secretion pressure that pushes the sweat through the microfluidic channel not only without any contact with the environment but also without any blocking of the sweat glands that may alter perspiration; 3) the device is biocompatible and safe from a cytotoxicity point of view;^82^ 4) the sensing range must cover the sweat concentrations expected during sports practice (ca. from 1 to 25 mM and even higher);^83^ 5) sweat must be transported by means of the microfluidics design in a stable and expeditious manner to minimize the delay time of the sensors; 6) the sensor must have a fast response time, high sensitivity, and selectivity toward lactate; 7) the calibration task should be minimized and easily accessed/understood by the end-user; and 8) the on-body measurements and validation method must be accurate and resilient.

To meet all of the above-mentioned requirements, the use of electrochemical sensors has taken over the field at the time of writing, mainly due to their simplicity, low cost, high versatility, and great capacity for implementation into wearable devices.^84^ Despite the many great advantages of electrochemical sensors, a definitive solution for wearable sweat lactate sensors is still not available. At least one or more conditions from those listed above cannot be adequately met, and therefore the veracity of the provided information still remains questionable. Nevertheless, several recent high-quality publications have appeared in the field, and we are therefore sure that the challenge will be soon overcome. Particularly in regards to the validation issue, any mismatch between on-body measurements and the gold standard technique may arise from the technique used for sweat collection compromising the accuracy of the results.^5,85^ Currently, the use of adsorbent pads seems to be the most common strategy for such a purpose.^45,46,78^

Among the concepts explored for sweat lactate sensing, enzyme-based sensors with amperometric readout seem to lead in the vast majority of publications. A very interesting concept is based on the use of Prussian Blue (PB) material in combination with lactate oxidase (LOx) enzyme layering.^32^ Essentially, LOx converts lactate to pyruvate and hydrogen peroxide (H_2_O_2_), and the H_2_O_2_ subproduct is then detected in combination with the redox behavior of the PB, which is activated at a mild applied potential.^32^ Thus, the registered current changes according to the formed H_2_O_2_ concentration in the electrode, i.e., amperometry readout. This concept has successfully been implemented in different wearables.

In regard to accumulative type measurements, Cheng et al. reported on a textile-based wearable device capable of reading up to 5 mM lactate concentration in sweat via punctual data acquisition.^86^ Wider ranges of response were also reported when a diffusion layer is added on top of the enzyme. This is the case of the wrist device reported by Javey and co-workers (linear range of response from 5 to 30 mM)^26^ and the skin-worn (tattoo) sensor investigated by Imani et al. (3–20 mM),^87^ among others. The strategy recently proposed by Crespo and co-workers (see the previous section)^45,46,78^ combines an outer plasticized polymeric membrane to get a response from 1 to 30 mM while using true continuous measurements rather than accumulative ones. To date, the implementation of an outer layer in the sensor that is able to control the diffusion of the lactate from the sample to the enzyme is the most successful strategy followed to reach the concentration range expected for lactate in sweat.

Other strategies for lactate sensing involve the use of tetrathiafulvalene as the redox mediator rather than the PB (from 1 to 20 mM),^11,88^ grafted-polymerized MgO-templated carbon,^89^ and also nonenzymatic approaches comprising classical impedimetric,^90^ voltammetric,^91^ and potentiometric^92^ techniques. While impedimetric and voltammetric measurements only allowed for discrete lactate observations, continuous profiles may be accessible with a potentiometric readout. However, to the best of our knowledge, the displayed range of response does not cover lactate levels in sweat.^92^

Our main conclusion after analyzing all of these (carefully selected) works is that yet more efforts are necessary toward the realization of a wearable sensor providing accurate (i.e., appropiate successful validation), continuous, and real-time measurements of lactate in sweat. Seemingly, and after carefully inspecting the literature, the device recently reported by Crespo and co-workers is the one closest to such a challenge.^78^

## Sweat Lactate and Sports Physiology

In view of the lack of wearable sensors demonstrating accurate on-body measurements of sweat lactate, the reader may anticipate that most of the physiological observations connected to sweat lactate during sports performance were achieved via sampling and centralized analytical measurements. In this respect, the review by Derbyshire et al. provides a very comprehensive overview of the literature about sweat lactate studies from 1934 to 2012.^13^ The paper analyzed nine studies that investigated the relationship between sweat lactate and exercise intensities that cause a rise in blood lactate above the threshold. Interestingly, the review concluded that there were conflicting results. Five out the nine studies found a relationship between sweat lactate concentration and exercise intensity (increase or decrease in sweat lactate concentration with increasing exercise intensity), whereas the other four studies revealed no effect of exercise intensity on sweat lactate concentration at all. The overall message of the review was that blood lactate is not cleared by the sweat glands and that raising blood lactate levels does not affect sweat lactate concentration.

The authors also stated that the rate of sweating has a marked effect on the measurements: as the intensity of exercise increases, this will lead to higher sweating rates with concurrent dilution of lactate in sweat. In the reviewed studies, sweat was usually collected locally by absorbent materials or the Macroduct collector. The collection lasted for 2–20 min, and the samples were analyzed afterward with enzymatic lab-based methods.^24,93−96^ In the work by Mitsubayashi et al., the sweat was collected by scraping Petri dishes over the entire body, which resulted in a positive relationship between sweat lactate and exercise intensity.^97^ In general lines, different collection and analysis methods provided very distinct results and observations.

In the period from 1992 to 2021, our own revision of the field has revealed a total of 10 studies considering the task of sweat lactate sensing based on sampling methods. Four out of 10 studies found an inverse relationship between sweat lactate and exercise intensity,^14,98−100^ whereas one study showed inconclusive results;^101^ and five studies revealed a positive relationship between sweat lactate and exercise.^33,102−105^ These controversial results can, again, be explained by the different sweat collection methods, with some of them even using sweat stimulation before the exercise practice. On the other hand, we have come to realize that all the studies that found a positive lactate-exercise relationship used different techniques to eliminate the influence of sweat rate on the measurements. Some examples are local sweat stimulation before exercise (iontophoresis),^33,103^ wetting of a sweat sensor with sweat droplets,^102^ the use of a plastic container with an ethanol solution that came in contact with the skin surface for 60s,^104^ and sweat collection instantly after sauna or exercise.^105^ The rest of the studies used the corresponding sweat collection method (i.e., absorbent patch,^46^ wound dressing,^49^ sweat pouch,^48^ or paper filter^51^) during a certain (and perhaps relatively long) period of time (from 1 to 20 min), which seems to cause a dilution effect on sweat lactate due to increasing sweat rate with increasing time and/or exercise intensity.

**Figure 6:**
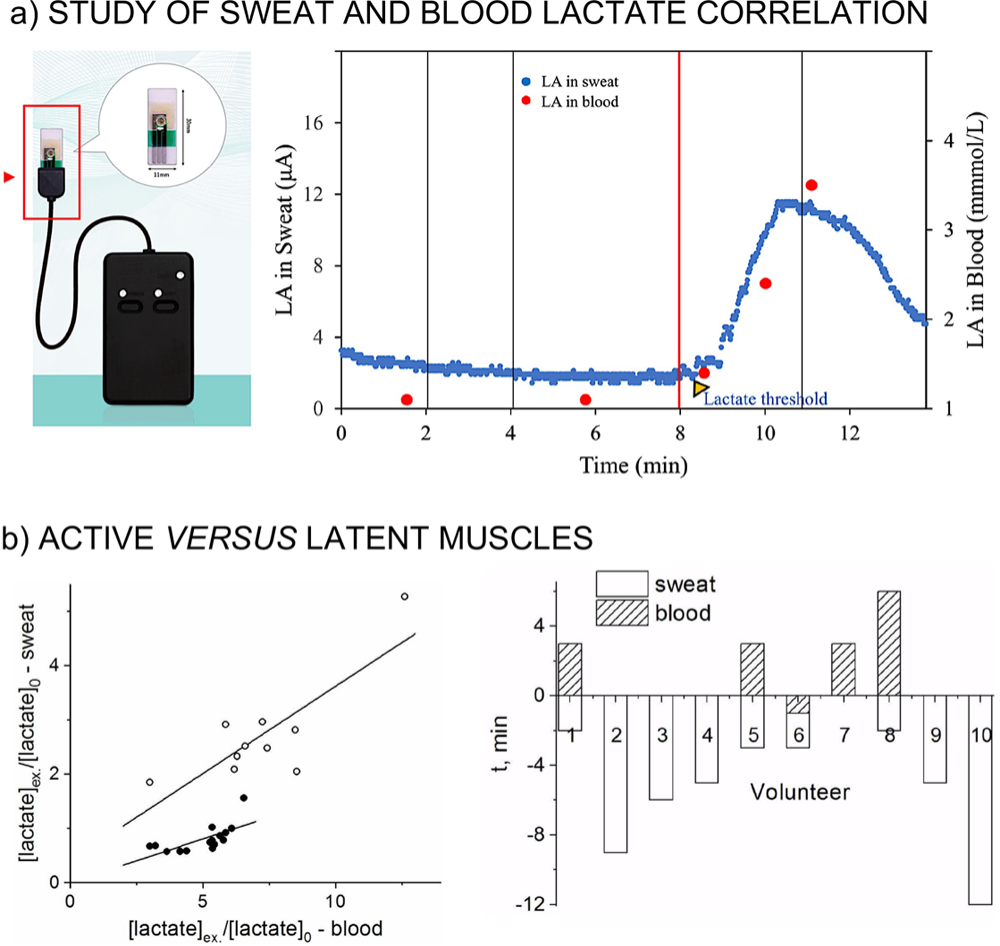
(a) Monitoring of sweat lactate (blue dots) and blood lactate (red dots) during incremental exercise. Reproduced with permission from ref (12) under a Creative Commons Attribution 4.0 International License (http://creativecommons.org/licenses/by/4.0/). **(b) Left:** Correlations between sweat and blood lactate observed in sweat from the thigh (white circles) and from the arm (black circles). **Right:** Time taken to achieve the maximum concentration of lactate in sweat in the working muscle and blood. Adapted from ref (33). Copyright 2020 John Wiley and Sons.

Since 2013 until the time of writing, a great number of papers based on sweat lactate measurements by means of wearable sensors have been published.^11,12,26−32,34^ However, to the best of our knowledge, all these devices work either on sweat accumulation principles or microfluidics based on large sweat reservoirs that result in the on-body observations at any time being an average of lactate sweat content from the beginning of the exercise practice to the time of measurement. Interestingly, a positive correlation between sweat lactate and exercise intensity has been reported in all of these papers. For example, the results reported by Seki et al. considered the monitoring of an anaerobic threshold in healthy subjects and patients.^12^ As can be seen in Figure 6a, a similar trend was found for sweat and blood lactate levels during exercise. Whether the findings of these papers are considered to be accurate or not, the use of sweat lactate as a proxy to blood should be possible, as well as using it to determine “lactate thresholds” and define training zones.^12,106^ Other hopes for sweat lactate wearable sensors, in contrast to blood measurements, are the real-time tracing of training intensity, effective feedback during training, and the definition of training load.^28,31,34^ The applications range from elite sports to recreational athletes who want to optimize their training as well as preventing over- or undertraining and injuries.^28,31,107^ It is crucial to validate the veracity of all the reported observations made to date with accurate sweat lactate measurements provided by a wearable sensor, working on a microfluidics principle rather than accumulative approaches.

Also, recent studies in the field have suggested that the scrutinized body area indeed affects the physiological observations.^33^ In more detail, the relationship between sweat and blood lactate levels during exhaustive physical exercise (cycling) in working (leg) and latent (arm) muscles (after pilocarpine electrophoresis sweat stimulation) was investigated. The study revealed an increase in sweat lactate concentration simultaneous with the increase in blood lactate in working muscles and no increase in sweat lactate concentration in the latent muscles, as shown in Figure 6b. Moreover, if sweat lactate is measured in an active muscle, this reflects the lactate threshold. Other studies positioned the wearable lactate sensor close to latent muscle sites during cycling (i.e., forehead,^12,26,28,30^ upper end lower arm,^12,26,31,32^ and lower back^27^) and observed the general trend of slightly increasing sweat lactate concentration with increasing exercise intensity. However, none of these studies examined active muscles. Further research considering different measurement sites is needed to confirm differences in sweat lactate evolution. It could be possible that only active muscles are to be monitored to track sports performance. We anticipate that this sort of information would be very valuable for the understanding of the lactate mechanism(s) in the body.

## Other Threshold Devices Used in Sports Physiology

Among all the useful contributions that precise sweat lactate observations may provide to the physiological domain, there are some hints indicating that sweat lactate may be a promising tool to monitor sports performance. If this is true, wearable lactate sensors will have to compete in the market with other wearable devices that are claimed for monitoring sports performance. In this context, the sensors most commonly used are based on physical parameter measurements, such as heart rate sensors and power meters, rather than chemical information. For example, analysis conducted from nonlinear dynamics of Heart Rate Variability (HRV) has been used to gain insights into the complex cardiovascular regulation during endurance-type exercise and define thresholds and training zones.^108,109^ This can be easily measured with commercially available heart rate monitors in combination with the so-called HRV logger app.^110^

Muscle oxygen saturation (SmO2) is a localized measure of muscle oxidative metabolism and can be acquired continuously and in a noninvasive manner by means of near-infrared spectroscopy (NIRS) methods. In the past, NIRS systems were expensive, cumbersome, fiber coupled devices, with their use limited to laboratory settings; but recently, low cost, wireless, and wearable devices have been developed and made commercially available with the aim of SmO2 monitoring. Some examples are the Humon,^111^ Moxy Monitor,^112,113^ and BSXinsight.^114^ These devices are used in sports to monitor oxygen saturation of the working muscles and calculate the “lactate threshold”.^111,114^ Interestingly, it was found that neither a SmO2 absolute threshold value nor relative threshold drop could identify the lactate threshold power accurately.^111^ Therefore, algorithms to estimate the lactate threshold that showed good agreement with the blood lactate have been developed.^111^ Although these are promising results, there are large variations in the measurements at the higher activity intensities,^112^ and these are indirect calculations of lactate thresholds. Some of the commercial devices are currently no longer on the market, which reflects the still vivid controversies in the field.

Sweat droplets can also be used to measure sweat lactate with a handheld device using potentiometric measurements.^102^ The device is commercially available, and the method is very similar to the handheld blood lactate devices where you need a small drop of blood to measure lactate. The concept is based on a nonequilibrium potentiometric measurement performed by disposable, chemically modified, screen printed carbon electrodes (SPCEs) that can be wetted with sweat during the exercise. The sweat lactate concentration changes during the exercise, reflecting the intensity of physical effort. Unfortunately, there are no continuous measurements available for this device, and it is not wearable.

## Conclusions

With the further development of wearable sensors, the possibility to accomplish sweat lactate measurements in a noninvasive way and continuously tracing its evolution while doing sports seems to be rather close to our hands. Significant efforts have been reported up to know. In contrast to the literature reported in the prewearable technology era, in which the analyses of sweat lactate and exercise intensities were rather contradictory, the recent evidence seems to be more consistent. Nevertheless, there are very few original studies with sweat wearable lactate sensors that include significant on-body data, with the vast majority of them showing some positive correlation between sweat and blood lactate. Unfortunately, most of the data were collected from chemical sensors that operate in an accumulative way, and thus, it is difficult to draw conclusions in relation with the sports physiology responsible for any of the reported lactate behaviors. All in all, we can conclude that sweat wearable lactate sensors are positioned as good candidates for sports physiology assessment. Furthermore, this new technology is not influenced from traditional issues related to sweat handling (evaporation, sweat rate, and skin surface contamination), and more accurate data are hence expected; but this is not enough, and researchers must provide undeniable evidence of possible correlations with blood lactate and other clinical biomarkers. It is also crucial to understand if there is any relationship between sweat lactate and sports performance, even exploring active and passive muscles in the physical activity. Many researchers in the field, physiologists, and sports clinicians have expressed that whether the relevance and applicability of sweat lactate can be demonstrated would mean a high contribution not only in terms of markers of performance (muscle status), health (nutritional and hydration), and recovery (inflammation, injury, and muscle damage) but also in providing data to build accurate models that explain with more confidence the origin of lactate in sweat. Finally, real-time sweat lactate measurements accompanied by other biomarkers may allow training sessions to be exhaustively monitored and adjusted. This can ensure that athletes do not train too hard or too little, thus providing the right training stimulus to enable the athlete to perform better in the long term. In this way, both top and recreational athletes will be able to train more efficiently, improve their performance, and prevent overload or injuries.
